# Aquaponics production of catfish and pumpkin: Comparison with conventional production systems

**DOI:** 10.1002/fsn3.1512

**Published:** 2020-04-08

**Authors:** Sunday Abraham Oladimeji, Victor Tosin Okomoda, Samuel Olabode Olufeagba, Shola Gabriel Solomon, Ambok Bolong Abol‐Munafi, Korede Isaiah Alabi, Mhd Ikhwanuddin, Chukwumeka Onwuka Martins, Joshua Umaru, Anuar Hassan

**Affiliations:** ^1^ Agricultural Department National Biotechnology Development Agency (NABDA) Abuja Nigeria; ^2^ Department of Fisheries and Aquaculture College of Forestry and Fisheries University of Agriculture Makurdi Nigeria; ^3^ Institute of Tropical Aquaculture and Fisheries Research (AQUATROP) Universiti Malaysia Terengganu Kuala Nerus Malaysia; ^4^ Faculty of Food Science and Fisheries Universiti Malaysia Terengganu Kuala Nerus Malaysia; ^5^ Department of Agricultural Extension and Management Federal College of Forestry Jos. Plateau Nigeria; ^6^ Faculty of Forestry Universiti Putra Malaysia Serdang Malaysia; ^7^ Fisheries Technology Department College of Agriculture Lafia Nigeria

**Keywords:** aquaculture, *Clarias gariepinus*, hydroponics, *Telfairia occidentalis*

## Abstract

Aquaponics is known to be a smart way of producing fish and crops simultaneously; however, there is a paucity of information about the extents of this system's efficiency over other conventional methods of food production. Thus, this study was designed to evaluate the performance of a catfish–pumpkin aquaponics system in comparison with recirculatory and static aquaculture systems (for fish performance), as well as irrigated and nonirrigated systems (for pumpkin performance). Results obtained showed that the production of fish in the aquaponics system was 29% and 75% more efficient than recirculatory and static aquaculture systems, respectively. The survival of the fish was also significantly improved probably due to better water quality in the aquaponics system. With respect to pumpkin production, yield in the aquaponics system was about five times the performance in irrigated land and eleven times those in nonirrigated land. This study gives definitive evidence to support the efficiency of the aquaponics system over other conventional food production methods.

## Graphical abstract

Aquaponics system was 29% and 75% more efficient than recirculatory and static aquaculture systems, respectively. Pumpkin yield in the aquaponics system was about five times the performance in irrigated land. Pumpkin yield in the aquaponics system was about eleven times the performance in nonirrigated land.

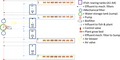

## 1 INTRODUCTION

Global consumption of fish is expected to hit 146 million tons annually by 2030; yet, supply is staggering low (FAO, 2008). This is despite the fact that aquaculture is the fastest growing animal food production sector in the world, accounting for about 50% of all fish products consumed by humans (FAO, 2016). Generally, the challenges associated with the food production sector in the twenty‐first century are nothing other than the world's population explosion which is exponential compared to the level of food production. Consequently, the increased demand for food is intensifying the pressures on natural resources and ecosystems as efforts are made to fill the supply gap (Delaide et al., 2017; Suhl et al., 2016). To efficiently solve these challenges, sustainable food production inched on low carbon imprints, less water, and a low land requirement is obvious the way forward for the future. This is because traditional aquaculture production systems in ponds (static systems) have a high water budget and cause significant negative environmental impacts (i.e., nutrient load of wastewater) (Klinger & Naylor, 2012; Verdegem, 2013).

Also, traditional crop production required large portions of land and high water budget and sometimes lead to deadly resource‐use conflict between farmers and herdsmen (Ajuwon, 2004; Fasona & Omojola, 2005; Udo, Ier, & Yemi, 2019). The need to adopt efficient production systems has paved the way for the development of urban farming methods such as Recirculatory aquaculture systems (RAS) and hydroponic systems. These closed systems are based on the concept of water reuse, hence has less water budget and causes less environmental impacts compared to conventional agriculture systems (Timmons, Ebeling, Wheaton, Summerfelt, & Vinci, 2010; Verdegem, 2013). However, there is still a need for water exchange in the RAS to reduce nitrogen waste accumulation below levels that could be toxic to fish (Rakocy & Hargreaves, 1993; Yildiz et al., 2017). Hydroponic system, on the other hand, strives on constant supplementation of nutrients to meet the requirement of the crops grown (Blidariu & Grozea, 2011; Love et al., 2015; Pantanella, Cardarelli, Colla, Rea, & Marcucci, 2010; Pulvenis, 2016; Sonneveld & Voogt, 2009).

Aquaponics production system is therefore considered one of the most efficient and environmentally sustainable farming methods of the twenty‐first century (FAO, 2014; Oladimeji, Olufeagba, Ayuba, Solomon, & Okomoda, 2020) as it combined the RAS with hydroponic system, hence mitigating the adverse effects of these methods on the environment (Tyson, Treadwell, & Simonne, 2011; Zou, Hu, Zhang, Guimbaud, et al., 2016). This integration ensures that the nitrogen‐rich fish wastes produced are utilized as organic fertilizer by the plant (Blidariu & Grozea, 2011; Love et al., 2015; Pantanella et al., 2010), while the purified wastewater recycled from the plant is used in rearing the fish (Zou, Hu, Zhang, Xie, et al., 2016). The aquaponics system is therefore an innovative, reliable and cost‐effective way of boosting food production as well as mitigating communal clashes for land use. However, there is a paucity of information establishing the efficiency of this system over other conventional methods of fish and vegetable production. This study is designed to fill that gap of knowledge.

The choice of a catfish and pumpkin for this study is predicated on the importance of both commodities. The African catfish *Clarias gariepinus* (Burchell, 1822) is one of the most emblematic and important freshwater aquaculture species in Africa and South‐East Asia (Okomoda, 2018; Okomoda, Koh, & Shahreza, 2017; Solomon, Okomoda, & Ochai, 2013). Pumpkin *Telfairia occidentalis* is a member of the Cucurbitaceae family indigenous to Southern Nigeria and grown mainly for its leafy vegetables and seeds (Akoroda, 1990). The leaves have antioxidants, hepatoprotective, and antimicrobial properties (Nwanna, 2008), while the seeds have a high proportion of oil (Akoroda, 1990). The *T. occidentalis* is therefore primarily exploited for food (i.e., soup making), herbal medicines, and as a potential export commodity (Nwanna, 2008; Okoli & Mgbeogu, 1983). Catfish production and pumpkin production have historically been through conventional means. Only recently, we reported our findings on the performance of a catfish–pumpkin aquaponics system using different grow beds (Oladimeji et al., 2020). The concept of the aquaponics system is also not popular in many underdeveloped/developing countries of the world. It is hoped that the findings of this study will buttress the efficiency of the aquaponics system over conventional planting/fish rearing methods.

## 2 MATERIALS AND METHOD

**Figure 1:**
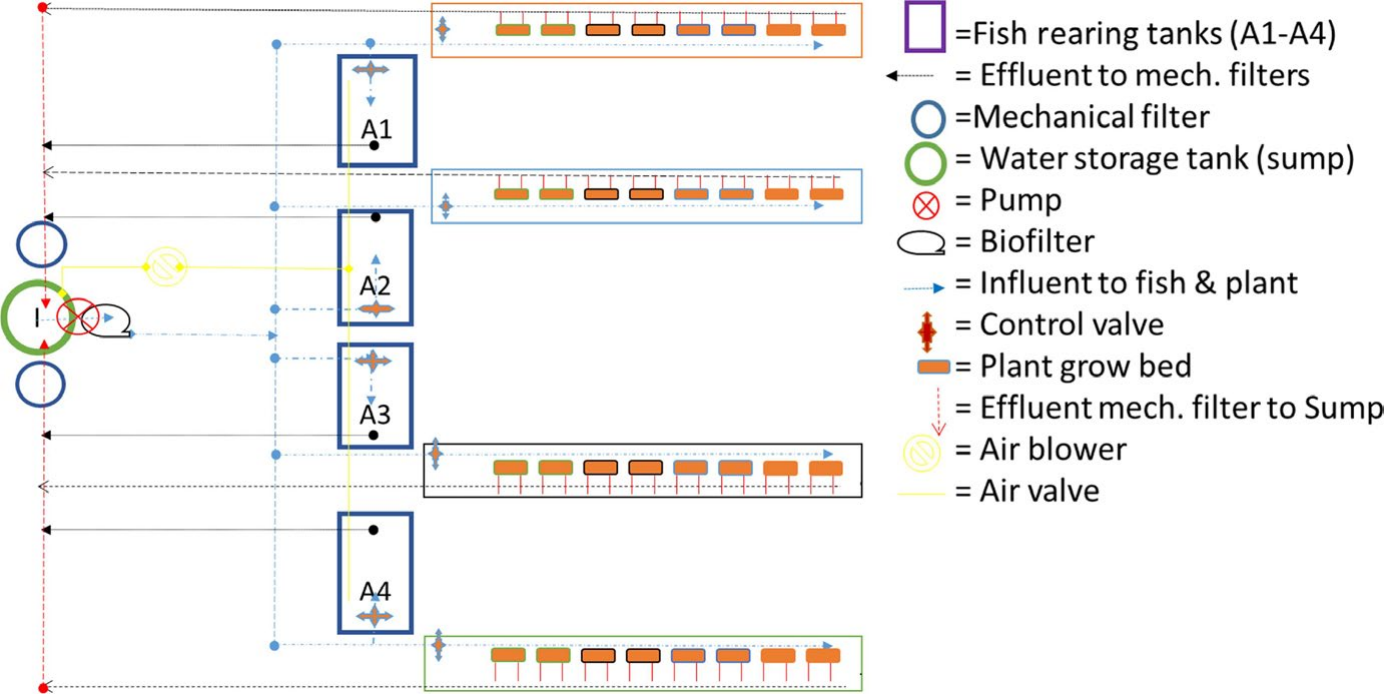
Aquaponics system layout as used in this study (adapted from Oladimeji et al., 2020)

The study was conducted at the Agricultural Department of the National Biotechnology Development Agency (NABDA) Headquarter located along the Umaru Musa Yar'adua Express, Airport road Lugbe Abuja, Nigeria. The study area is situated at latitude 9°16′N and longitude 7°20′E, and 300 m altitude above sea level with 1,500 mm rainfall annually. The catfish–pumpkin aquaponics system used was according to the specification previously reported by Oladimeji et al. (2020) as shown in Figure 1 and Table 1 (in a glasshouse). The grow bed used was periwinkle shell; this has been earlier demonstrated to be better for pumpkin production (Oladimeji et al., 2020). The pumpkin pods for this study were obtained from a known source in Eastern Nigeria (Imo state), while the juveniles of African catfish *C. gariepinus* were obtained from the NABDA aquaculture production facility.

**Table 1:** System dimension of the aquaponics system (adapted from Oladimeji et al., 2020)

S/N	Tanks	Dimensions
1	Fish rearing tanks	200 L
2	Planting bowls	0.045 m^3^
3	Mechanical filter tank	20 L
4	Sump tank	250 L
5	Hydraulic loading rate	7.5 L/hr
6	Water volume in the system	800 L (0.8 m^3^)
7	RAS land area occupied	12 m^2^
8	The hydroponic land area occupied	48 m^2^

Static aquaculture systems used in this study were composed of four numbers of tanks installed just behind the glasshouse for the aquaponics system. Water change was done once every week according to Okomoda, Tiamiyu, and Iortim (2016) who earlier reported this to be better for the growth of the African catfish. Similarly, the Recirculatory aquaculture system (RAS) was installed alongside the static system and was a replica of the RAS of the aquaponics system. Both the RAS and the aquaponics system were daily‐added water at a level of 5% of the total water which is to compensate for evaporation and transpiration losses, respectively (Maucieri et al., 2017). All the quadruplicate tanks used to raise the fish in the different setup were 200 L each and water level maintained three‐quarter mark throughout the study period. Also, 50 randomly selected juveniles (mean weight = 10.01 ± 0.11 g; stocking density = 0.25 g/L) were stock in each rearing tanks in all the systems.

The conventional farming in this study was done one meter left and right of the glasshouse facility holding the aquaponics system (each 48 m^2^ by size). The soil properties (physical and chemical) were tested at the soil science laboratory of the University of Agriculture Makurdi and found suitable for the growth of pumpkin (Table 2). Both the irrigated land and the nonirrigated land received rainwater throughout the study period; however, grow beds of the aquaponics system were shielded due to the glasshouse installation and only received water from the RAS connected to it. The land used for irrigation was fed pond water from the static aquaculture system every week when a water change is done. The pods of pumpkin in this study were first cut to expose the seeds and then planted in pairs. Thirty‐two seeds of pumpkin were planted in each planting system at the rate of one pair per troughs of the aquaponics system and one pair per 0.045 m^3^ of the irrigated land and nonirrigated land.

**Table 2:** Physical and chemical properties of the soil for irrigated and nonirrigated land (at 0–15 cm depth)

S/no.	Mechanical composition	Value
1	Clay (g/kg)	8.64
2	Silt	25.33
3	Sand	65.47
4	Textural classification (USD)	Loamy sand

	Chemical composition	
1	pH (H_2_O)	5.55
2	pH (0.01 M CaCl_2_)	5.03
3	T N%	0.15
4	Avail. P (mg/kg)	16.08
5	K (mg/kg)	0.14
6	C (mg/kg)	0.53
7	Mg (mol/kg)	3.12
8	Ca (mol/kg)	4.16
9	Na (mol/kg)	82.71
10	CEC (mol/kg)	2.71

During the course of the study, the African catfish in all the systems were fed commercial diet Coppens^®^ (45% CP; 1.5% fiber; 8.2% moisture, and 9.5% ash) at a rate of 5% body weight. The weights of the fish were taken weekly using a sensitive weighing balance (0.001 g) and the feeding regime adjusted as appropriate. At the end of the study which lasted for 4 months, growth performance and other indices were done as adopted by Okomoda, Tiamiyu, and Akpan (2017), Okomoda, Koh, et al. (2017) and shown below.

1. Growth rate (g/d) = $\frac{W_{2}-W_{1}}{t_{2}-t_{1}}$

where *W*_1_ = initial weight (g); *W*_2_ = final weight (g); *t*_2_ − *t*_1_ = duration between *W*_2_ and *W*_1_ (days).

- Specific growth rate (%/day) = $\frac{\log_{e}\left(W_{2}\right)-\log_{e}\left(W_{1}\right)}{t_{2}-t_{1}}$
- Feed conversion ratio (FCR) = $\frac{\text{dry feed intake}}{W_{2}-W_{1}}$
- Feed efficiency ratio (%FER) = $\frac{(W_{2}-W_{1})\times 100}{\text{dryfeed intake}}$
- %Survival = $\frac{\text{fish stocked}-\text{mortality}}{\text{fish stocked}}\times 100$

Water samples were collected from the fish tanks in the various system and tested for temperature, pH, dissolved oxygen (DO), ammonia (NH_3_), nitrite‐nitrogen (NO_2_), and nitrate‐nitrogen (NO_3_) using a digital multiparameter water checker (Hanna water tester Model HL 98126) and chemical water kits.

In the course of this study, some assumptions were made and could have constituted the core of limitations for the current study: Firstly, it was assumed that the number of fish reared and vegetable seedling propagated in the aquaponics unit matches nutrient input and requirements for the smooth running of the system. Secondly, it was thought that a hydraulic loading rate of 7.5 L/hr was sufficient for the aquaponics setup in this study. Thirdly, it was also assumed that the daily addition of 5% of water to both the RAS and the aquaponics system was sufficient to compensate losses through evaporation and transpiration losses, respectively. Fourthly, it was assumed that the plants in the irrigated and nonirrigated land got required nutrients from the soil without any need to add any form of fertilizer (similar to practices of indigenous pumpkin farmers). Also, the authors assumed the nonrearing of the crops under aquaponics conditions did not affect the performance of the fish since the routine procedure of weeding and other needed husbandry requirement were given. Lastly, it was assumed that the rainfall during the study period was sufficient for the growth of pumpkin in the conventional system.

Data collection for the yield parameters of the plants was initiated 4 weeks after seed germination and subsequently every 2 weeks according to the method specified by Cornelissen et al. (2003). The parameters collected include vine length, leave numbers, number of branches, and plant yield. Data were analyzed using Minitab 14 computer software. Firstly, descriptive statistics of all data were done followed by a one‐way analysis of variance (ANOVA). When significant (*p* < .05) differences were observed, Fisher's least significant difference was used to separate the means.

It is also important to state that the experimental protocols for this study were reviewed and approved by the National Biotechnology Development Agency (NABDA) committee on research. More so, all methods used in this study involving the care and use of animals were in accordance with international, national, and institutional guidelines.

## 3 RESULT AND DISCUSSION

The water quality parameters of the rearing tanks in the aquaponics system and RAS (Table 3) were within the recommended range for aquaculture (Ajani, Akinwole, & Ayodele, 2011; FEPA, 1988) but not for the static system. The levels of dissolved oxygen and nitrogen waste in the static system could be implicated in the performance of fish as observed in this study (Table 4). Boyd (1982) had opined that dissolved oxygen should be above 5 mg/L to support the survival and development of aquatic life in any culture system. However, many fishes have been reported to tolerate much lower. The study by Ostrand and Wilde (2001) had earlier shown that cyprinids are tolerant to the dissolved oxygen concentration of about 2.1 mg/L. Similarly, Okomoda, Koh, Hassan, Amornsakun, and Shahreza (2019) observed that African catfish *C. gariepinus* could survive in dissolved oxygen below 1 mg/L because of its accessory respiratory organ. Although no standard of Ammonia has been reported particularly for the rearing of *C. gariepinus,* many studies have reported varying recommendations. According to Knepp and Arkin (1973), levels above 0.2 mg/L are toxic to fish. Somervilla, Cohen, Pantanella, Stankus, and Lovatelli (2014), however, suggested a level less than 1 ppm, while Ridha and Cruz (2001) recommends 0.02 mg/L. Akinwole (2005), on the other hand, had recommendations of <8.8 mg/L for warmwater fish culture. The observations in this study, however, may have resulted from the combined effect of increased un‐ionized ammonia in the absence of oxygen. This has been proven to be detrimental to African catfish survival in a static system over a prolonged period of time (Okomoda et al., 2019).

**Table 3:** Water quality parameters from three different culture systems for fish

Parameters	Aquaponics system	Recirculatory system	Static system	*p*‐value
Temp (°C)	27.83 ± 0.20	28.08 ± 0.02	28.55 ± 0.22	.131
DO (ppm)	5.23 ± 0.03^a^	4.97 ± 0.04^b^	3.34 ± 0.01^c^	.001
pH	6.80 ± 0.05^a^	6.85 ± 0.03^a^	6.03 ± 0.11^b^	.003
NH_3_ (mg/L)	0.05 ± 0.003^c^	0.82 ± 0.002^b^	3.34 ± 0.023^a^	.001
NO_2_ (mg/L)	0.27 ± 0.04^c^	0.68 ± 0.02^b^	2.23 ± 0.01^a^	.001
NO_3_ (mg/L)	0.32 ± 0.02^b^	1.64 ± 0.09^a^	0.33 ± 0.03^b^	.001

Note

Mean in the same row with different superscripts differs significantly (*p* < .05).

**Table 4:** Growth parameters of fish reared in three different culture systems

Parameters	Aquaponics system	Recirculatory system	Static system	*p*‐value
Initial wt (g)	9.99 ± 0.21	9.92 ± 0.26	10.21 ± 0.26	.822
Final wt (g)	685.25 ± 7.02^a^	538.8 ± 17.8^b^	398.8 ± 5.08^c^	.001
Wt gain (g)	671.12 ± 6.82^a^	529.5 ± 9.04^b^	373.1 ± 3.40^c^	.001
Growth rate (g/day)	6.03 ± 0.06^a^	4.72 ± 0.16^b^	3.72 ± 0.02^c^	.001
SGR	7.55 ± 0.01^a^	7.04 ± 0.04^b^	6.40 ± 0.03^c^	.003
FCR	1.09 ± 0.05^c^	1.27 ± 0.03^b^	2.55 ± 0.05^a^	.012
FCE (%)	86.51 ± 0.68^a^	78.82 ± 1.89^b^	49.18 ± 0.38^c^	.009
Survival (%)	94.25 ± 2.12^a^	80.60 ± 1.20^b^	59.24 ± 1.91^c^	.009

Note

Mean in the same row with different superscripts differs significantly (*p* < .05).

The high value of ammonia recorded in the static system could be linked to the high stocking density used and nonfrequent water renewal as done in the other closed systems (RAS and aquaponics). Although nitrate‐nitrogen (NO_3_‐N) and nitrite (NO_2_) are products of ammonia oxidation, only the latter is considered to be of serious concern in fish culture (Ebeling, Losordo, & Delong, 1993; Timmons, Ebeling, Wheaton, Summerfelt, & Vinci, 2002). Nitrite is toxic as it can lead to prompt fish fatality. Toxic levels prevent the spread of oxygen within the bloodstream of fish (Bernstein, 2011). Throughout our study period, NO_2_ concentrations were lower than the sublethal concentration of 2.83 mg/L reported by Dabrowska and Własow (1986), and Thangam (2014). It is noteworthy that values recorded for the static system surpass the nitrite standard suggested by Somervilla et al. (2014), while that of the closed system was within the standard (i.e., <1 ppm). Nitrite (NO_2_) level from the aquaponics system was much lower than the other systems probably because of the double‐sided action of nitrobacteria present in the biological filters and the grow beds for the pumpkins in the system. The value of NO_3_, however, was higher in the RAS owing to the build‐up of nitrate by nitrobacteria present without corresponding usage. Plants utilize nitrates for growth (Britto, Herbert, & Konzucker, 2002). According to Syafiqah et al. (2015), plants in the aquaponics system act as biological filters, thereby absorbing nutrients such as nitrate and NH_3_ from the system. This, therefore, explains the low levels observed in the aquaponics system in our study. The above‐mentioned is in line with the findings of Hambrey (2013) and Wahyuningsih, Effendi, and Wardiatno (2015) who observed that leafy vegetables (lettuce) significantly decrease nitrogen waste such as NH_3_ and NO_3_ in aquaponics system for up to 92% and 50%, respectively. A similar observation was also made by Oladimeji et al. (2020) when the inlet water of the aquaculture system was compared to its effluent water in a catfish–pumpkin aquaponics setup. The observation of low NO_3_ in the static system, however, may have resulted from a reduced nitrification process in this system.

**Figure 2:**
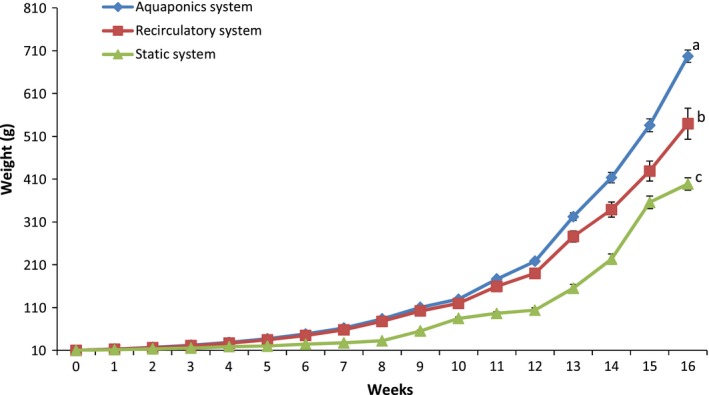
Weight of *Clarias gariepinus* raised in the different aquaculture systems over 16 weeks. Data shown are mean ± *SE*

**Figure 3:**
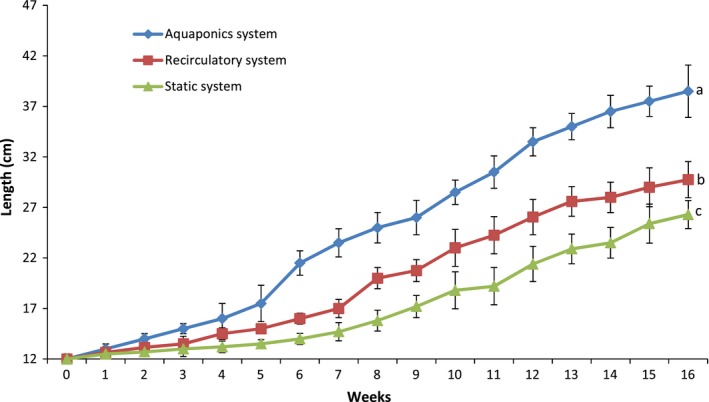
Length of *Clarias gariepinus* raised in the different culture systems over 16 weeks. Data shown are mean ± *SE*

One of the advantages of aquaponics is the unilateral input of nutrients from the fish feed into the system. Hence, feed does not only serve as a nutrient source for the fish, but also, indirectly, for the plants as well (Goddek et al., 2015; Rakocy, Bailey, Shultz, & Thoman, 2004; Rakocy, Masser, & Losordo, 2006; Savidov, Hutchings, & Rakocy, 2005). This means in terms of input cost for optimum performance, growing fish, and pumpkin in the aquaponics system is much lesser than conventional means. This is the same position of Hochman, Hochman, Naveh, and Zilberman (2018) as they observed that the introduction of aquaponics system diversified farmers' sources of income, increasing the yield of fish and plant over other forms of food production systems. In line with this finding, this study showed that fish grow better in the aquaponics system recording 29% and 75% efficiency than growth in the recirculatory and static aquaculture systems, respectively (Table 4; Figures 2 and 3). Obviously, the performance difference in the fish in the different systems can be linked to water quality since the same feed and similar environment were used. Ajani et al. (2011) had noted that fish continuously exposed to more than 0.2 mg/L of the un‐ionized form of ammonia exhibited reduced growth and increased susceptibility to disease. This may explain the reduced growth in the RAS and the static system compared to the aquaponics system in this study. Aquaponics production of fish in this study was better than the reports of Palm, Bissa, and Knaus (2014) for Nile Tilapia *Oreochromis niloticus* and African catfish *C. gariepinus* grown with a low‐tech closed ebb‐flow substrate aquaponics system. The differences in our study with these reference studies may be linked to many factors which include the type of aquaponics system, the stocking density of the fish and species differences.

**Figure 4:**
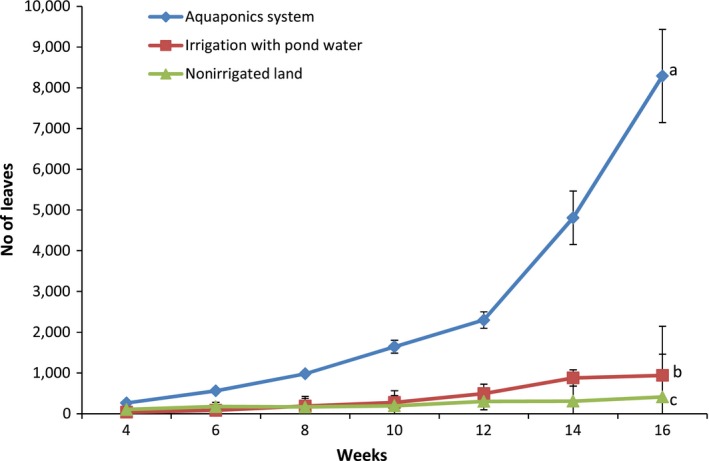
Leave numbers of pumpkin propagated in the different systems. Data shown are mean ± *SE*

**Figure 5:**
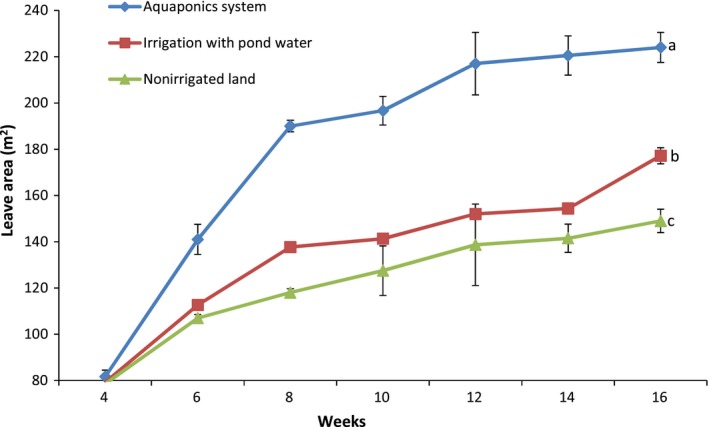
Leave areas of pumpkin propagated in the different systems. Data shown are mean ± *SE*

**Figure 6:**
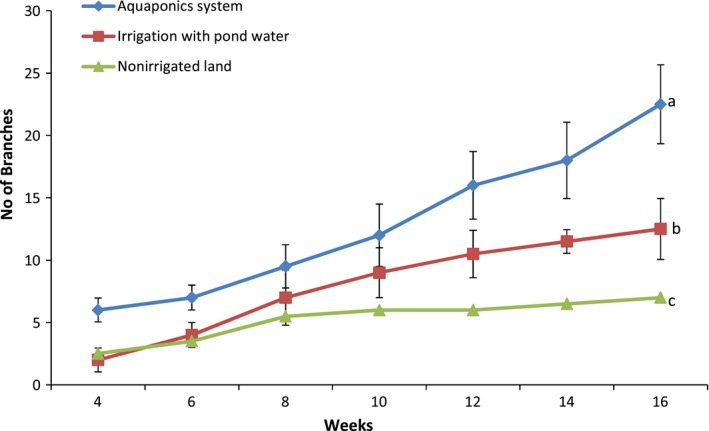
Number of branches of pumpkin propagated in the different systems. Data shown are mean ± *SE*

**Figure 7:**
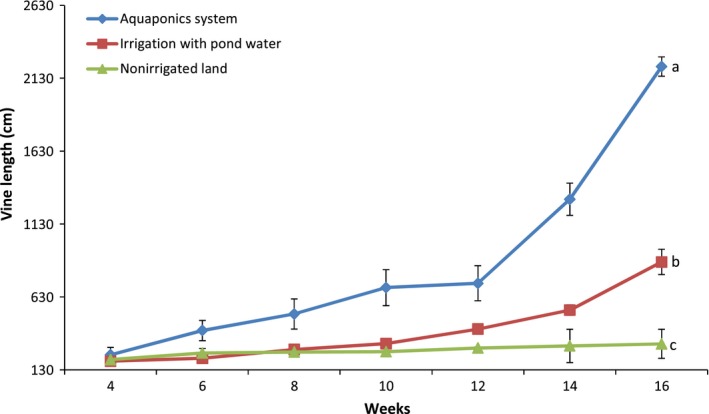
Pumpkin vine length in the different systems. Data shown are mean ± *SE*

**Figure 8:**
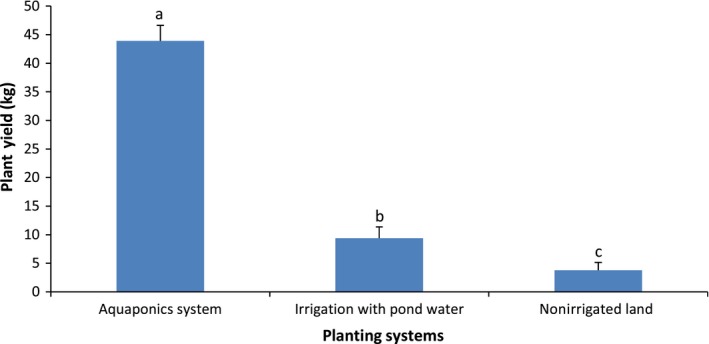
Pumpkin yield in the different systems. Data shown are mean ± *SE*. Bars with different letters are significantly different from each other (*p* ≤ .05)

Pumpkin production in the aquaponics system was five times the performance in irrigated land and eleven times those in nonirrigated land as observed for plant characteristics and overall yield (Figures 4, 5, 6, 7, 8). Aquaponics had earlier been heralded not only for its suitability for environments with limited land and water but also for its ability to produce three to six times the vegetables in conventional planting systems (Resh, 2004). According to Roosta and Hamidpour (2011), Liang and Chien (2015), factors such as nutrient availability and ease of uptake influence the production and harvestable biomass of crops. Similarly, Maucieri et al. (2019) concluded that water characteristics, together with nutrient availability, affected many characteristics of vegetable production especially its final yield. Possibly, the higher levels of nutrient availability for plants in the aquaponics system and those planted in the irrigated lands (from the static aquaculture system) resulted in their better yield and plant parameters compared to the nonirrigated land. It is also noteworthy that the pumpkin in the different systems did not show any observable signs of disease infection. Although no study exists for the comparative performance of aquaponics and irrigated/nonirrigated lands, our findings are similar to those of Goddek and Vermeulen (2018) who observed that lettuce grown in the commercial aquaculture‐based hydroponic system had enhanced growth performance compared to those grown in the conventional hydroponic nutrient solution on a commercial scale. Earlier findings of Delaide, Goddek, Gott, Soyeurt, and Jijakli (2016) had also revealed the same under laboratory conditions. Their study claimed that aquaponic‐grown lettuce in significantly similar chemical nutrient solutions shows better growth advantage and yield of approximately 40% over performance in a conventional hydroponic system. However, contrary to the trend in these studies, the findings by Suhl et al. (2016) on tomato showed that performance was similar in the aquaponics system and hydroponic system without any significant production advantage. This study has given substantial evidence to support the claim that the aquaponics system is more efficient in the production of catfish and pumpkin compared to other production systems. Future studies can test this hypothesis on a commercial scale.

## ETHICAL APPROVAL

The authors declare that no fund was received for the conduct of this research; hence, there is no conflict of interest what so ever (financial or otherwise) to declare. The authors declare that the experimental protocols and procedures were ethically reviewed and approved by the “National Biotechnology Development Agency (NABDA) ethical committee on research.” More so, all methods used in this study involving the care and use of animals were in accordance with international, national, and institutional guidelines. This includes compliance with the US National Research Council's Guide for the Care and Use of Laboratory Animals, the US Public Health Service's Policy on Humane Care and Use of Laboratory Animals, and Guide for the Care and Use of Laboratory Animals.
